# Novel Cross-domain Symbiosis between *Candidatus* Patescibacteria and Hydrogenotrophic Methanogenic Archaea *Methanospirillum* Discovered in a Methanogenic Ecosystem

**DOI:** 10.1264/jsme2.ME22063

**Published:** 2022-11-12

**Authors:** Kyohei Kuroda, Kengo Kubota, Shuka Kagemasa, Ryosuke Nakai, Yuga Hirakata, Kyosuke Yamamoto, Masaru K. Nobu, Takashi Narihiro

**Affiliations:** 1 Bioproduction Research Institute, National Institute of Advanced Industrial Science and Technology (AIST), 2–17–2–1 Tsukisamu‐Higashi, Toyohira‐ku, Sapporo, Hokkaido, 062–8517 Japan; 2 Department of Frontier Sciences for Advanced Environment, Graduate School of Environmental Studies, Tohoku University, 6–6–06 Aza-Aoba, Aramaki, Aoba-ku, Sendai, Miyagi 980–8579, Japan; 3 Department of Civil and Environmental Engineering, Graduate School of Engineering, Tohoku University, 6–6–06 Aza-Aoba, Aramaki, Aoba-ku, Sendai, Miyagi 980–8579, Japan; 4 Bioproduction Research Institute, National Institute of Advanced Industrial Science and Technology (AIST), Central 6, Higashi 1–1–1, Tsukuba, Ibaraki 305–8566, Japan

**Keywords:** Candidate Phyla Radiation (CPR) superphylum, *Candidatus* Patescibacteria, 32-520/UBA5633, *Methanospirillum*, cross-domain symbiosis

## Abstract

To identify novel cross-domain symbiosis between *Candidatus* Patescibacteria and Archaea, we performed fluorescence *in situ* hybridization (FISH) on enrichment cultures derived from methanogenic bioreactor sludge with the newly designed 32-520-1066 probe targeting the family-level uncultured clade 32-520/UBA5633 lineage in the class *Ca.* Paceibacteria. All FISH-detectable 32-520/UBA5633 cells were attached to *Methanospirillum*, indicating high host specificity. Transmission electron microscopy observations revealed 32-520/UBA5633-like cells that were specifically adherent to the plug structure of *Methanospirillum*-like rod-shaped cells. The metagenome-assembled genomes of 32-520/UBA5633 encoded unique gene clusters comprising pilin signal peptides and type IV pilins. These results provide novel insights into unseen symbiosis between *Ca.* Patescibacteria and Archaea.

The *Candidatus* Patescibacteria/Candidate Phyla Radiation (CPR) superphylum is a major bacterial phylogenetic group that includes various uncultivated lineages (Brown *et al.*, 2015; Parks *et al.*, 2018). *Ca.* Patescibacteria are known for their small cell (*e.g.*, average diameter of ~0.2‍ ‍μm) (He *et al.*, 2021) and genome (*e.g.*, 1.1±0.2‍ ‍Mbp) sizes (Tian *et al.*, 2020; Kagemasa *et al.*, 2022), and genomic traits suggest that most *Ca.* Patescibacteria require external nutrient sources through symbiotic interactions (*e.g.*, commensal, mutualistic, and parasitic relationships) due to a lack of biosynthesis genes (Castelle *et al.*, 2018; Fujii *et al.*, 2022).‍ ‍Although previous studies demonstrated *Ca.* Patescibacteria-Bacteria interactions through cultivation-based approaches (He *et al.*, 2015; Moreira *et al.*, 2021; Yakimov *et al.*, 2022), cross-domain symbiosis was only reported for *Ca.* Patescibacteria-eukarya (Gong *et al.*, 2014). In our recent study, we successfully verified novel symbiosis between *Ca.* Patescibacteria and Archaea; *i.e.*, *Ca.* Yanofskybacteria/UBA5738 of the class *Ca.* Paceibacteria (formerly known as *Ca.* Parcubacteria/OD1 [Brown *et al.*, 2015]) specifically parasitized with aceticlastic methanogenic archaea *Methanothrix* (Kuroda *et al.*, 2022b) (Kuroda, K., *et al.*, 2022. Symbiosis between Patescibacteria and Archaea discovered in wastewater-treating bioreactors. *bioRxiv* https://doi.org/10.1101/2022.04.10.487813) using anaerobic wastewater treatment bioreactor sludge samples as seed materials for cultivation (Kuroda *et al.*, 2022a). These findings implied that unseen cross-domain symbiosis remains to be found in the methanogenic environment. In‍ ‍the present study, using a previously reported *Ca.* Patescibacteria-enrichment culture (Kuroda *et al.*, 2022b), we discovered novel and different cross-domain symbiosis between *Ca.* Patescibacteria and a hydrogenotrophic methanogen by employing 16S rRNA-targeting fluorescence *in situ* hybridization (FISH), transmission electron microscopy (TEM), and genome ana­lyses.

In enrichment cultures, we used anaerobic granular sludge samples obtained from a bioreactor as the starting material with serial dilutions (10^–1^, 10^–3^, 10^–4^, and 10^–6^ defined as d1, d2, d3, and d4, respectively) and provided potential growth factors, such as yeast extract, amino acids, and nucleoside monophosphates for the growth of *Ca.* Patescibacteria (see Supporting information). By continuous cultivation, methane gas production was confirmed from some enrichment cultures (Kuroda, K., *et al.*, 2022. Symbiosis between Patescibacteria and Archaea discovered in wastewater-treating bioreactors. *bioRxiv* https://doi.org/10.1101/2022.04.10.487813). Through phase-contrast microscopic observations of previously reported *Ca.* Patescibacteria-enriched cultures (Kuroda *et al.*, 2022b), we detected one to five ultramicrobacterial cells (diameter <1‍ ‍μm) at the edge of rod-shaped cells (Fig. 1A and B). According to previous findings on *Ca.* Patescibacteria-Archaea cross-domain symbiosis, we predicted that these small coccoid cells were *Ca.* Patescibacteria based on high relative abundance (up to 18.5%) in enrichment cultures based on 16S rRNA gene amplicon sequencing (Table S1) (Kuroda *et al.*, 2022b). To confirm the phylogeny of small cells and juxtaposed rod-shaped cells, we performed FISH experiments targeting the domains Bacteria and Archaea, order *Methanomicrobiales*, and genus *Methanolinea* using the specific probes EUB338mix (Amann *et al.*, 1990; Daims *et al.*, 1999), ARC915 (Raskin *et al.*, 1994), MG1200 (Raskin *et al.*, 1994), and NOBI633 (Imachi *et al.*, 2008), respectively. We initially confirmed that rod-shaped cells hybridized with ARC915 and MG1200, but not with EUB338mix or NOBI633, suggesting that these cells were assigned to *Methanospirillum*, while small cells that attached to *Methanospirillum* were EUB338mix-positive bacteria predicted to be *Ca.* Patescibacteria (Fig. S1 and S2). We then newly designed a 32-520-1066 probe targeting two bacterial OTUs (OTU0014 and OTU0072) associated with uncultured clade 32-520 in the class *Ca.* Paceibacteria, which was the second most predominant patescibacterial population in the culture systems (Fig. 1C, Table S2 in Supporting information, and Table S3). As a result, small coccoid cells juxtaposed with rod-shaped *Methanospirillum* cells belonged to uncultured clade 32-520 (Fig. 1D, E, and F). All FISH-detectable 32-520-1066-positive cells (>20 micrographs) were specifically attached to *Methanospirillum* cells with no‍ ‍free-living active cells, indicating high host-symbiont specificity between *Methanospirillum* and the 32-520 lineage. According to FISH imaging, 32-520-associated *Methanospirillum* cells showed low fluorescence intensity/ribosomal activity, indicating parasitism, which was similar to the symbiotic interaction between *Methanothrix* and *Ca.* Yanofskybacteria/UBA5738 (Kuroda *et al.*, 2022b). In enrichment cultures, *Methanolinea* formed multicellular filamentous morphologies (Fig. S2G), which were previously observed in syntrophic propionate-degrading enrichment cultures due to low hydrogen partial pressure (Imachi *et al.*, 2008); therefore, *Methanolinea* in cultures may associate with free living syntrophic bacteria, presumably *Syntrophomonas*-related OTUs (up to 4.5%).

TEM revealed sub-micron 32-520-like cells (0.42±0.09‍ ‍μm in length and 0.28±0.04‍ ‍μm in width) adhering to rod-shaped cells with a plug structure that closely resembled *Methanospirillum* (Beveridge *et al.*, 1985; Firtel *et al.*, 1993) (Fig. 2A, B, C, D, E, and F). Although it was not confirmed in the TEM photographs obtained in the present study, *Methanospirillum* was previously shown to possess flagella (also known as archaella) at the plug structure (Poweleit *et al.*, 2017; Albers and Jarrell, 2018). These findings suggest that the 32-520 lineage recognizes and attaches to the cell surface structure of *Methanospirillum* localized at the cell poles and then inhibits cell elongation, division, and metabolism. 32-520-like small cells were consistently surrounded by extracellular polymeric substances (EPS) or pilin-like substances and specifically attached to the plug structure of *Methanospirillum* cells (Fig. 2A, B, C, D, E, and F), suggesting the importance of these substances for attachment and/or growth. Furthermore, 32-520-like organisms that appeared to be in the cell division stage were attached to *Mehtanospirillum* plug structures (Fig. 2A, B, C, D, E, and F); therefore, these 32-520-like cells were in the active growth phase in culture media, but not in the spore-forming-like dormant state. Calculated cell volumes (0.0208±0.0105‍ ‍μm^3^) were smaller than 0.1‍ ‍μm^3^ (*i.e.*, ultramicrobacterial [Nakai, 2020]).

In previous studies, gene arrays containing small signal peptides were found in members of *Ca.* Patescibacteria, which are considered to be relevant to parasitism (McLean *et al.*, 2020; Kuroda *et al.*, 2022b). To identify gene arrays, we analyzed the metagenome-assembled genomes of 32-520 lineages PMX.108 and PMX.50 (reconstructed genomes in a previous study [Kuroda *et al.*, 2022b] that encode nearly full-length 16S rRNA gene sequences with 100% similarities to OTU0014 and OTU0072, respectively). Based on the phylogenetic classification through SILVA v138.1 and GTDB r207 taxonomies, PMX.108 and PMX.50 belonged to the 32-520 lineage and family UBA5633 of the order *Ca.*‍ ‍Paceibacterales, respectively (Fig. 1C and 3A). Both genomes lacked several biosynthetic pathways, such as amino acids and fatty acid biosynthesis (Tables S4 and S5), suggesting a host-dependent lifestyle. Based on a homology search with the blastp platform (Altschul *et al.*, 1990), highly similar gene clusters containing multiple genes encoding pilin signal peptides and components of type IV pilin (*pilBCDMT*) were found in the PMX.108 and PMX.50 genomes (Fig. 3B, Tables S4, S5, and S6). Type IV pilin plays important roles in surface/host cell adhesion and parasitism (Giltner *et al.*, 2012). To further clarify the distribution of gene clusters, we performed a homology search with other lineages belonging to the family UBA5633. The gene array was mostly conserved in the family UBA5633 (Fig. 3B and Table S7). These results suggest the potential of members of this family for symbiosis with *Methanospirillum* by employing pilin signal peptides/type IV pilin gene clusters.

In summary, we successfully cultivated/enriched lineage 32-520/UBA5633 in the class *Ca.* Paceibacteria and discovered novel and second cross-domain symbiosis between *Ca.* Patescibacteria/CPR and the hydrogenotrophic methanogen *Methanospirillum*. Microscopic observations and genome ana­lyses clearly indicated symbiosis between lineage 32-520/UBA5633–*Methanospirillum* as host-specific parasitism with a gene cluster encoding pilin signal peptides and type IV pilin. Further cultivation combined with transcriptomic/proteomic experiments will provide more detailed insights into the overlooked ecology of *Ca.* Patescibacteria in methanogenic ecosystems (*e.g.*, anaerobic bioreactors).

## Citation

Kuroda, K., Kubota, K., Kagemasa, S., Nakai, R., Hirakata, Y., Yamamoto, K., et al. (2022) Novel Cross-domain Symbiosis between *Candidatus* Patescibacteria and Hydrogenotrophic Methanogenic Archaea *Methanospirillum* Discovered in a Methanogenic Ecosystem. *Microbes Environ ***37**: ME22063.

https://doi.org/10.1264/jsme2.ME22063

## Supplementary Material

Supplementary Material 1

Supplementary Material 2

## Figures and Tables

**Fig. 1.**
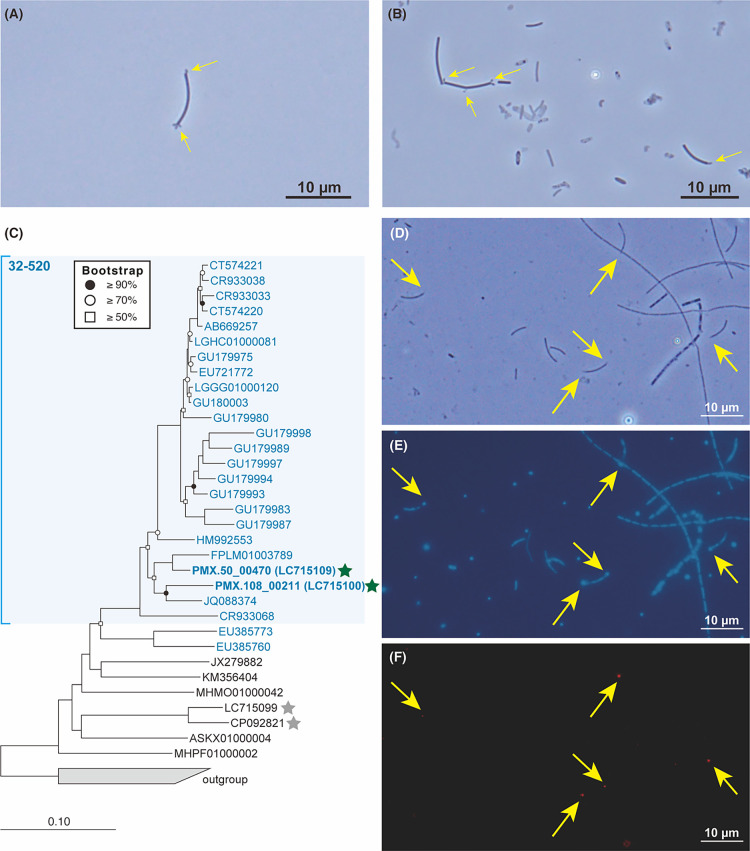
(A) and (B) Phase-contrast micrographs of small cells attached to rod-shaped cells in culture systems A-d2 on days 12 (A) and 23 (B). Yellow arrows indicate small cells attached to rod-shaped cells. (C) Phylogenetic tree of the 32-520 lineage based on 16S rRNA gene sequences. The 16S rRNA gene-based tree was constructed using the neighbor-joining method implemented in the ARB program. The OTUs obtained in the present study are shown in bold type in the tree. Sequences that match the 32-520-1066 probe are shown in blue font. Star symbols indicate the symbiotic partners of these lineages (green: *Methanospirillum* (this study) and gray: *Methanothrix* (Kuroda *et al.*, 2022b) (Chen, X., *et al.*, 2022. *Candidatus* Nealsonbacteria (OD1) in a methanogenic enrichment culture is likely an ectosymbiotic biomass recycler. *bioRxiv* https://doi.org/10.1101/2022.04.20.488981). Micrographs of (D) phase-contrast micrographs, (E) 4′,6-diamidino-2-phenylindole dihydrochloride staining, and (F) fluorescence *in situ* hybridization (FISH) by uncultured order-level clade 32-520 targeting the 32-520-1066-Cy3 probe obtained from the culture system B-d1-d1 on day 23. Yellow arrows indicate FISH-detectable 32-520 cells belonging to the class *Ca.* Paceibacteria (belonging to the phylum *Ca.* Patescibacteria).

**Fig. 2.**
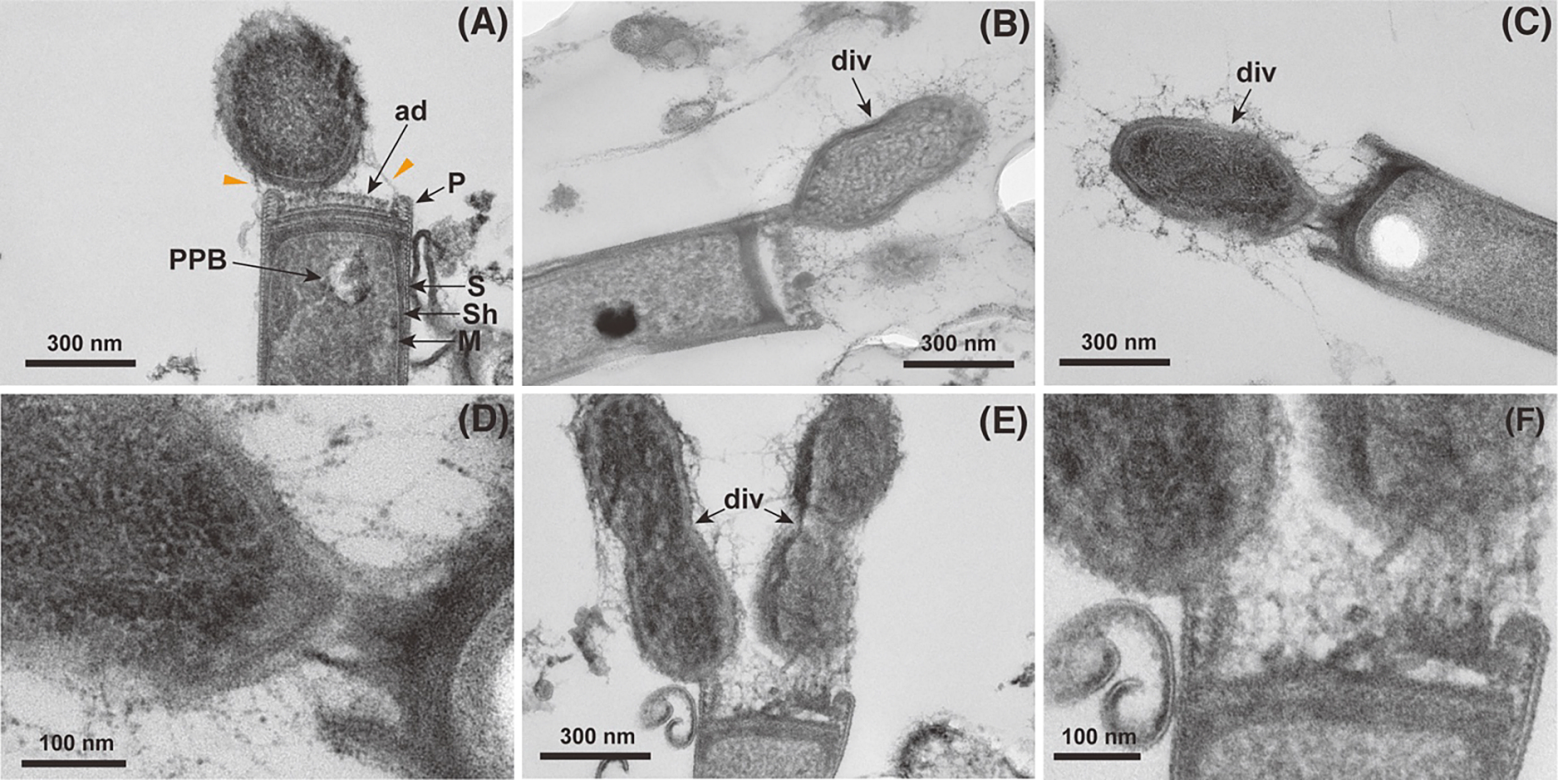
(A–F) Transmission electron micrographs of small coccoid sub-micron cells attached to *Methanospirillum*-like cells in culture system A-d2 on day 40. S, Sh, M, P, ad, div, and PPB indicate the S-layer, sheath structure, cell membrane, plug structure, adhesion substance, dividing cells, and polyphosphate-like body, respectively. Orange arrows indicate extracellular polymeric substances (EPS) or pilin-like substances.

**Fig. 3.**
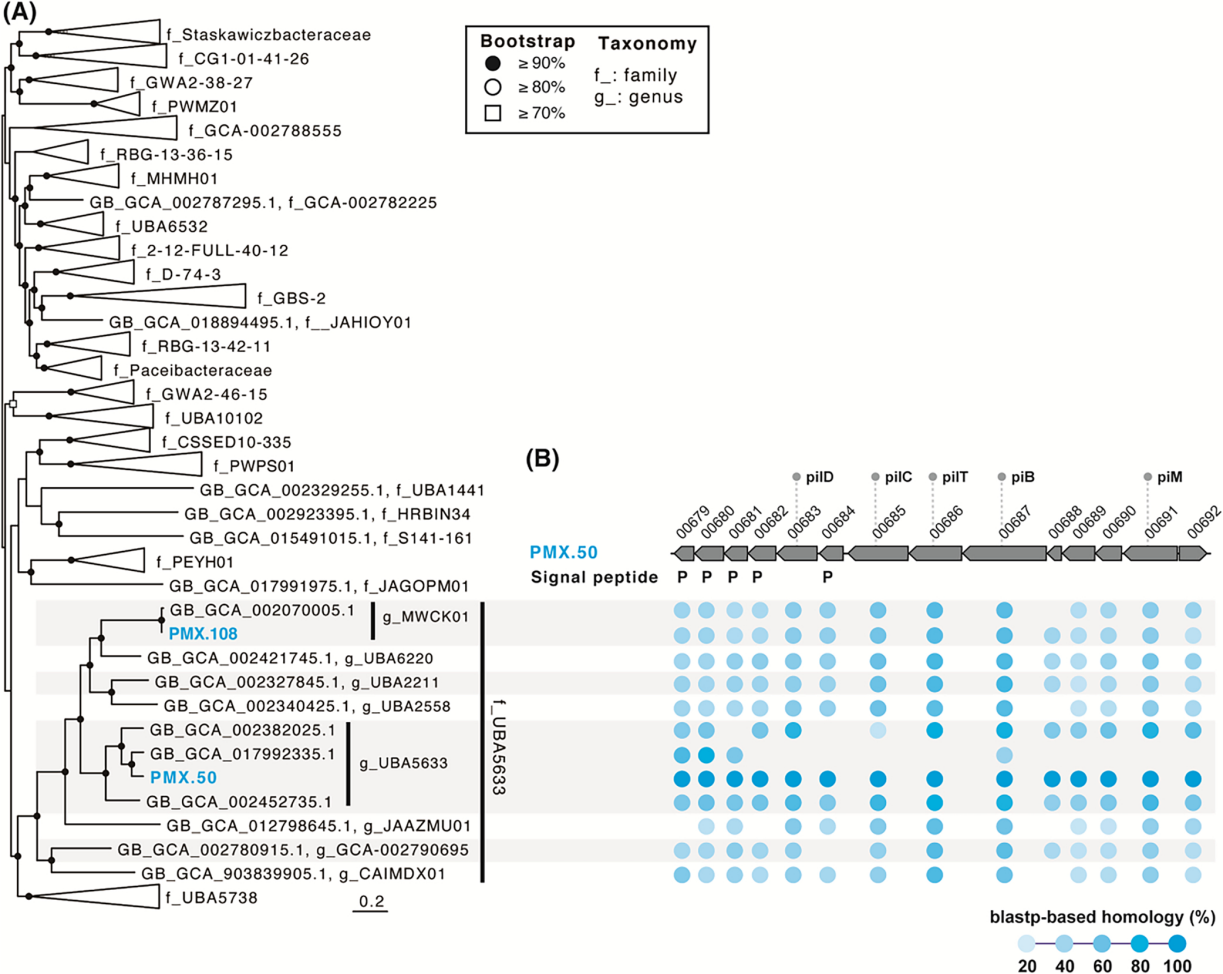
(A) Phylogenetic tree of the order *Ca.* Paceibacterales based on concatenated phylogenetic marker genes of GTDBtk 2.0.0 (ver. r207). The phylogenetic position of the metagenomic bins of PMX.50 and PMX.108 are shown in blue. (B) Gene clusters containing multiple genes with signal peptides in 32-520/UBA5633. P indicates the pilin signal peptide. Blue circles indicate a blastp-based homology (threshold ≤1e–10) with the metagenomic bin PMX.50. No annotated genes are hypothetical proteins (based on the annotation using BlastKOALA in Table S4). Abbreviated locus tags are shown in (B) (*e.g.*, “PMX.50_00679” as “00679” in the row of PMX.50).
