# Supplementary material for: Novel Cross-domain Symbiosis between Candidatus Patescibacteria and Hydrogenotrophic Methanogenic Archaea Methanospirillum Discovered in a Methanogenic Ecosystem
Source: Microbes Environ. 2022 Nov 12;37(4):ME22063. doi: 10.1264/jsme2.ME22063 (PMC9763046; doi:10.1264/jsme2.ME22063)
Supplement: Supplementary file 1 — Supplementary Material 1 [file 37_22063_s1.pdf]

Novel cross-domain symbiosis between *Candidatus* Patescibacteria and hydrogenotrophic methanogenic archaea *Methanospirillum* discovered in a methanogenic ecosystem

– Supporting information–

Kyohei Kuroda[1*], Kengo Kubota[2,3], Shuka Kagemasa[1,3], Ryosuke Nakai[1], Yuga Hirakata[4], Kyosuke Yamamoto[1], Masaru K. Nobu[4], Takashi Narihiro[1*]

[1]Bioproduction Research Institute, National Institute of Advanced Industrial Science and Technology (AIST), 2-17-2-1 Tsukisamu-Higashi, Toyohira-ku, Sapporo, Hokkaido, 062-8517 Japan
[2]Department of Frontier Sciences for Advanced Environment, Graduate School of Environmental Studies, Tohoku University, 6-6-06 Aza-Aoba, Aramaki, Aoba-ku, Sendai, Miyagi 980-8579, Japan
[3]Department of Civil and Environmental Engineering, Graduate School of Engineering, Tohoku University, 6-6-06 Aza-Aoba, Aramaki, Aoba-ku, Sendai, Miyagi 980-8579, Japan
[4]Bioproduction Research Institute, National Institute of Advanced Industrial Science and Technology (AIST), Central 6, Higashi 1-1-1, Tsukuba, Ibaraki 305-8566, Japan

[*]Co-corresponding authors:
Takashi Narihiro, E-mail: t.narihiro@aist.go.jp
Kyohei Kuroda, E-mail: k.kuroda@aist.go.jp

Materials and Methods

Enrichment cultures used of this study were obtained from previous study (Kuroda *et al.*, 2022). We prepared three culture media as (A) 1m M acetate, 0.03% (w/v), 0.1 mM adenosine 5'-monophosphate, uridine 5'-monophosphate, guanosine 5'-monophosphate, and cytidine 5'-monophosphate, 1% (w/v) MEM Non-essential Amino Acids Solution (cat no. 139-15651, FUJIFILM Wako Pure Chemical Co. Ltd., Tokyo, Japan), 1% (w/v) MEM Essential Amino Acids Solution (cat no. 132-15641, FUJIFILM Wako Pure Chemical Co. Ltd., Tokyo, Japan), *Methanothrix soehngenii* GP6 (DSM 3671, 0.2 mL/20mL-medium), and *Methanosarcina barkeri* MS (DSM 800, 0.2 mL/20mL-medium) in inorganic medium. (B) A-medium without acetate, and (C) A-medium without yeast extract. We prepared 4th serial dilution (10^{-1}, 10^{-3}, 10^{-4}, and 10^{-6}) defined as d1–d4. To further enrich the targeting microorganisms, we transferred 2 mL of 1st dilution (defined as d1) of culture system B (defined as B-d1) and 2nd dilution of culture systems A (A-d2) and 3 (C-d2) into fresh medium (defined as A-d2-d1, B-d1-d1, and C-d2-d1) on days 33 under same substrates condition.

16S rRNA gene sequence analysis and probe design

One mL of cultivated microorganisms was collected from 1st-batch of each enrichment culture system on days 12 and 33 (A–C with 1st–3rd serial dilution) and 2nd-batch culture systems (A-d2-d1, B-d1-d1, and C-d2-d1) on days 23 and 32 and collected microbial cells by centrifugation at 17,750 g. DNA was extracted from microbial cells using FastDNA Spin Kit for Soil (MP Biomedicals, Santa Ana, California, USA) according to the manufacturer's protocol. The 16S rRNA gene sequence analysis and data analysis were performed according to previous study (Kuroda *et al.*, 2022). The phylogenetic tree of nearly full-length 16S rRNA gene sequences was constructed based on neighbor-joining methods in ARB version 7.0 (Ludwig *et al.*, 2004) using SILVA138.1 database for small subunit rRNA gene sequences. The sequences between *E. coli* position of 220 and 1460 were used for phylogenetic tree construction.

A probe specific for the 32-520 lineage was designed using the ARB software (Table S2). The newly designed 32-520-1066 probe specificity and coverage are shown in Table S3. The 32-520-1066 probe covers 95.7% (22/23 match) of 32-520 lineages as perfect match based on SILVA138.1 database using TestProbe 3.0 [https://www.arb-silva.de/search/testprobe/] and perfectly matches to 16S rRNA gene sequences of targeted 32-520 (LC715109 and LC715100) in the enrichment cultures (Figure 1C). Besides, the designed probe shows no perfect match for other detected *Ca*. Patescibacteria in all culture systems, which were confirmed by ARB software with SILVA138.1 database (Ludwig *et al.*, 2004). The designed probe sequence was subjected to mathFISH analysis [http://mathfish.cee.wisc.edu] (Yilmaz *et al.*, 2011) to predict hybridization efficiency. Specific formamide concentrations for the designed probe were experimentally determined by FISH experiments (described in **Fluorescence *in situ* hybridization** section in the Supporting information) at 0, 10, 15, 20, 25, and 30% as formamide concentrations. The specific signals of small coccoid cells juxtaposed with rod-shaped *Methanospirillum* cells were obtained in the enrichment cultures samples at all formamide concentration. At 30% formamide concentration, the FISH signals of small coccoid cells became weaker; therefore, we decided 25% formamide concentration as optimal condition for the 32-520-1066 probe.

Genome analysis

Metagenome-assembled genomes of 32-520 were obtained from previous study (Kuroda *et al.*, 2022). The taxonomic classification of the genomes was evaluated using GTDBtk v2.0.0 (GTDB release207; default parameters) (Chaumeil *et al.*, 2019). The genomes were annotated through a combination of Prokka v1.14.6 (Chaumeil *et al.*, 2019), BlastKOALA (Chaumeil *et al.*, 2019), and DRAM software (--use_uniref option with default setting) (Shaffer *et al.*, 2020) and manual annotation. Genomic tree was constructed using concatenated

phylogenetic marker genes of obtained bin and order *Ca.* Paceibactelales genomes. Conserved marker genes were identified using "gtdbtk identify" with default parameters and aligned to reference genomes using "gtdbtk align" with taxonomic filters (--taxa_filter o__Paceibacterales) (Chaumeil *et al.*, 2019). Phylogenetic tree was constructed using IQ-TREE version 2.1.4-beta (-B 1000) with automatically optimized substitution model (Q.yeast+F+R9) (Minh *et al.*, 2020). For the manual annotation, metagenomic bins were assigned using blastp version 2.6.0 to reference genomes of family UBA5633. Signal peptides of the genomes were annotated using SignalP 6.0 (Teufel *et al.*, 2022).

Fluorescence *in situ* hybridization

For fluorescence *in situ* hybridization (FISH) experiments, we used the 4% paraformaldehyde fixed samples obtained from 2nd-batch of enrichment culture systems A-d2-d1, B-d1-d1, and C-d2-d1 (cultivation days 23) (Kuroda *et al.*, 2022). Fluorescence *in situ* hybridization (FISH) was performed as described previously (Sekiguchi *et al.*, 1999). The hybridization and washing were carried out at 46ºC for 4–17 hours and at 48ºC for 20 mins, respectively. The FISH probes were shown in Table S2. An equimolar mixture (defined as EUB338mix) of EUB338 (Amann *et al.*, 1990), EUB338I, EUB338II, and EUB338III (Daims *et al.*, 1999) were used for detection of all bacteria. Formamide concentrations used in this study were follows: EUB338mix and NOBI633 (Imachi *et al.*, 2008), 10%; ARC915 (Raskin *et al.*, 1994), 35%; and MG1200 (Raskin *et al.*, 1994) and 32-520-1066, 25%. All probes were labeled with FITC, Cy3, or Cy5. The FISH samples were also stained with 4',6-diamidino-2-phenylindole dihydrochloride (DAPI). The microscopic images were observed by epifluorescence microscope (BX-53, Olympus, Japan) with a color CCD camera (DP-74, Olympus, Japan). Phase-contrast, FISH, and DAPI micrographic images were uniformly processed across the entire images using Adjust color of Preview application on Mac OS 11.6.5, Photoshop CC (Adobe Creative Cloud), and ImageJ 1.53k (Schneider *et al.*, 2012).

Table S2. Fluorescently labeled 16S rRNA-targeted oligonucleotide probes used in this study.

Probes	Target group	Probe sequence (5' to 3')	Reference
EUB338	Bacteria	GCTGCCTCCCGTAGGAGT	(Amann *et al.*, 1990)
EUB338I	Bacteria	GCAGCCTCCCGTAGGAGT	(Daims *et al.*, 1999)
EUB338II	Bacteria	GCAGCCACCCGTAGGTGT	(Daims *et al.*, 1999)
EUB338III	Bacteria	GCTGCCACCCGTAGGTGT	(Daims *et al.*, 1999)
ARC915	Archaea	GTGCTCCCCCGCCAATTCCT	(Raskin *et al.*, 1994)
MG1200	Methanomicrobiales	CGGATAATTCGGGGCATGCTG	(Raskin *et al.*, 1994)
NOBI633	*Methanolinea*	GATTGCCAGTTTCTCCTG	(Imachi *et al.*, 2008)
32-520-1066	32-520 in *Ca.* Paceibacteria	GAGCAACTCAAGCCACCTGCTG	This study

Transmission electron microscopy

On cultivation days 40, approximately 1 mL of cultivated media from culture system A-d2 was sampled and treated for transmission electron microscopy observation according to previous study (Kuroda *et al.*, 2022). Ultra-thin sections at 70 nm with a diamond knife using an ultramicrotome (Ultracut UCT, Leica, Vienna, Austria). The sections were stained with 2% uranyl acetate at room temperature for 15 mins and washed with distilled water followed by secondary-stained with Lead stain solutions (Sigma-Aldrich Co., Tokyo, Japan) at room temperature for 3 mins. The grids were observation by a transmission electron microscope (TEM) (JEM-1500Plus, JEOL Ltd., Tokyo, Japan) at 100 kV. Digital images were observed with a CCD camera (EM-14830RUBY2, JEOL Ltd., Tokyo, Japan). The cell diameters were measured from 8 single-cells attached on *Methanospirillum*-like cells.

The cell volumes were calculated according to Van Wambeke and Bianchi (1985) (Van Wambeke and Bianchi, 1985).

Deposition of DNA sequence data

The raw sequence data and binned metagenome data were deposited into the DDBJ Sequence Read Archive database (DRA013834 and DRA014327). The 16S rRNA gene sequences were deposited in the DDBJ/EMBL/GenBank databases (LC715096–LC715128).

References

Amann, R.I., Binder, B.J., Olson, R.J., Chisholm, S.W., Devereux, R., and Stahl, D.A. (1990) Combination of 16S rRNA-targeted oligonucleotide probes with flow cytometry for analyzing mixed microbial populations. *Appl Environ Microbiol* **56**: 1919–1925.

Chaumeil, P.-A., Mussig, A.J., Hugenholtz, P., and Parks, D.H. (2019) GTDB-Tk: a toolkit to classify genomes with the Genome Taxonomy Database. *Bioinformatics* **36**: 1925–1927.

Daims, H., Brühl, A., Amann, R., Schleifer, K.-H., and Wagner, M. (1999) The Domain-specific Probe EUB338 is Insufficient for the Detection of all Bacteria: Development and Evaluation of a more Comprehensive Probe Set. *Syst Appl Microbiol* **22**: 434–444.

Imachi, H., Sakai, S., Sekiguchi, Y., Hanada, S., Kamagata, Y., Ohashi, A., and Harada, H. (2008) Methanolinea tarda gen. nov., sp. nov., a methane-producing archaeon isolated from a methanogenic digester sludge. *Int J Syst Evol Microbiol* **58**: 294–301.

Kuroda, K., Yamamoto, K., Nakai, R., Hirakata, Y., Kubota, K., Nobu, M.K., and Narihiro, T. (2022) Symbiosis between Candidatus Patescibacteria and Archaea Discovered in Wastewater-Treating Bioreactors. *MBio* e0171122.

Ludwig, W., Strunk, O., Westram, R., Richter, L., Meier, H., Yadhukumar, et al. (2004) ARB: a software environment for sequence data. *Nucleic Acids Res* **32**: 1363–71.

Minh, B.Q., Schmidt, H.A., Chernomor, O., Schrempf, D., Woodhams, M.D., Von Haeseler, A., et al. (2020) IQ-TREE 2: New Models and Efficient Methods for Phylogenetic Inference in the Genomic Era. *Mol Biol Evol* **37**: 1530–1534.

Raskin, L., Stromley, J.M., Rittmann, B.E., and Stahl, D. a (1994) Group-specific 16S rRNA hybridization probes to describe natural communities of methanogens. *Appl Environ Microbiol* **60**: 1232–1240.

Schneider, C.A., Rasband, W.S., and Eliceiri, K.W. (2012) NIH Image to ImageJ: 25 years of image analysis. *Nat Methods* **9**: 671–675.

Sekiguchi, Y., Kamagata, Y., Nakamura, K., Ohashi, A., and Harada, H. (1999) Fluorescence In Situ Hybridization Using 16S rRNA-Targeted Oligonucleotides Reveals Localization of Methanogens and Selected Uncultured Bacteria in Mesophilic and Thermophilic Sludge Granules. *Appl Environ Microbiol* **65**: 1280–1288.

Shaffer, M., Borton, M.A., McGivern, B.B., Zayed, A.A., La Rosa, S.L., Solden, L.M., et al. (2020) DRAM for distilling microbial metabolism to automate the curation of microbiome function. *Nucleic Acids Res* **48**: 8883–8900.

Teufel, F., Almagro Armenteros, J.J., Johansen, A.R., Gíslason, M.H., Pihl, S.I., Tsirigos, K.D., et al. (2022) SignalP 6.0 predicts all five types of signal peptides using protein language models. *Nat Biotechnol* **40**: 1023–1025.

Van Wambeke, F. and Bianchi, M. (1985) Bacterial biomass production and ammonium regeneration in

Mediterranean sea water supplemented with amino acids. 2. Nitrogen flux through heterotrophic microplankton food chain. *Mar Ecol Prog Ser* **23**: 117–128.

Yilmaz, L.S., Parnerkar, S., and Noguera, D.R. (2011) mathFISH, a Web Tool That Uses Thermodynamics-Based Mathematical Models for In Silico Evaluation of Oligonucleotide Probes for Fluorescence In Situ Hybridization. *Appl Environ Microbiol* **77**: 1118–1122.

Supplementary Figures:

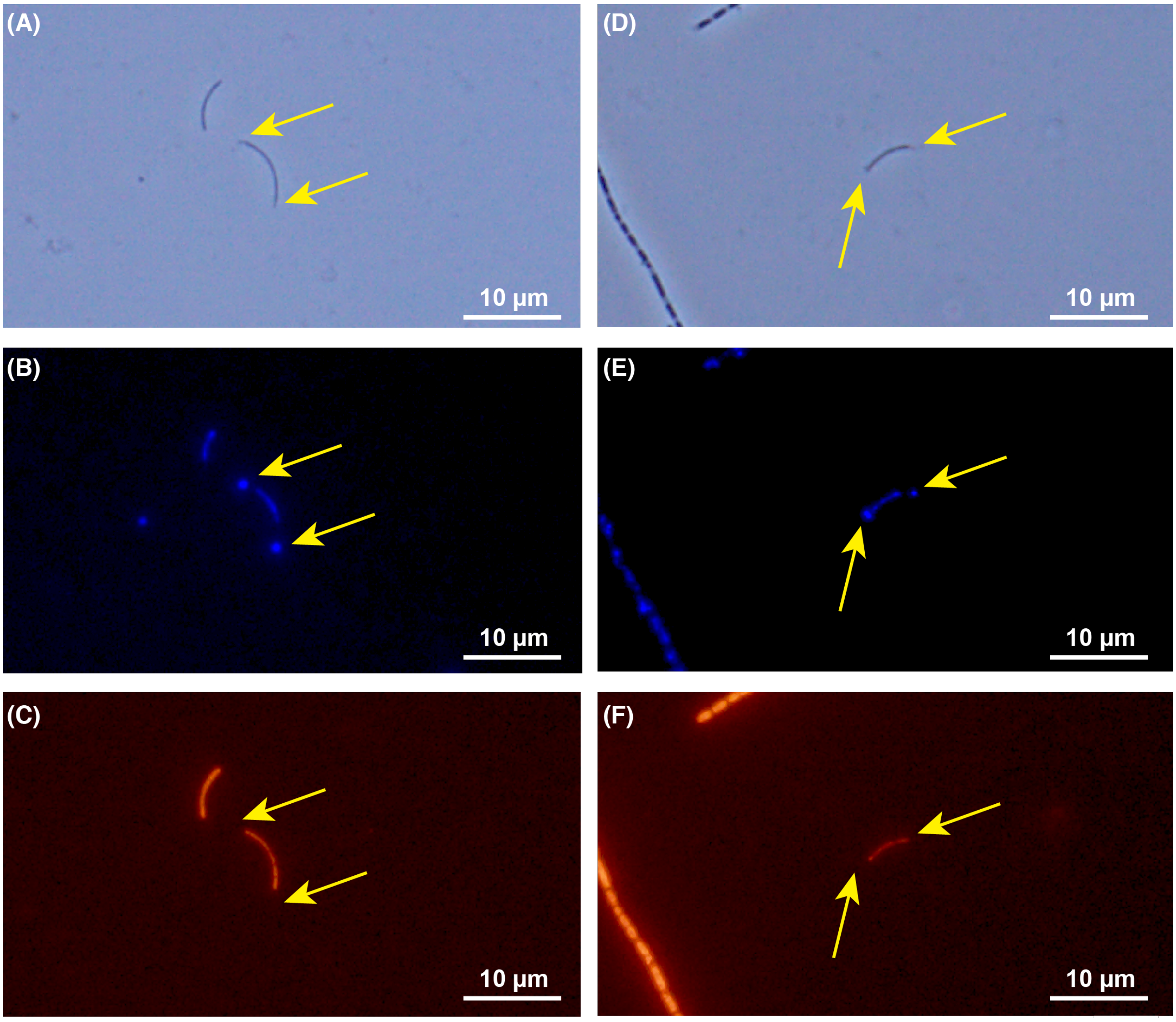

Figure S1 Micrographs of (A) and (D) Phase-contrast, (B) and (E) 4',6-diamidino-2-phenylindole dihydrochloride staining, and (C) and (F) fluorescence *in situ* hybridization targeting domain Archaea by ARC915-Cy3 probe obtained from culture system A-d2-d1 on cultivation days 23. Yellow arrows indicate attached microbial cells on the rod-shaped cells.

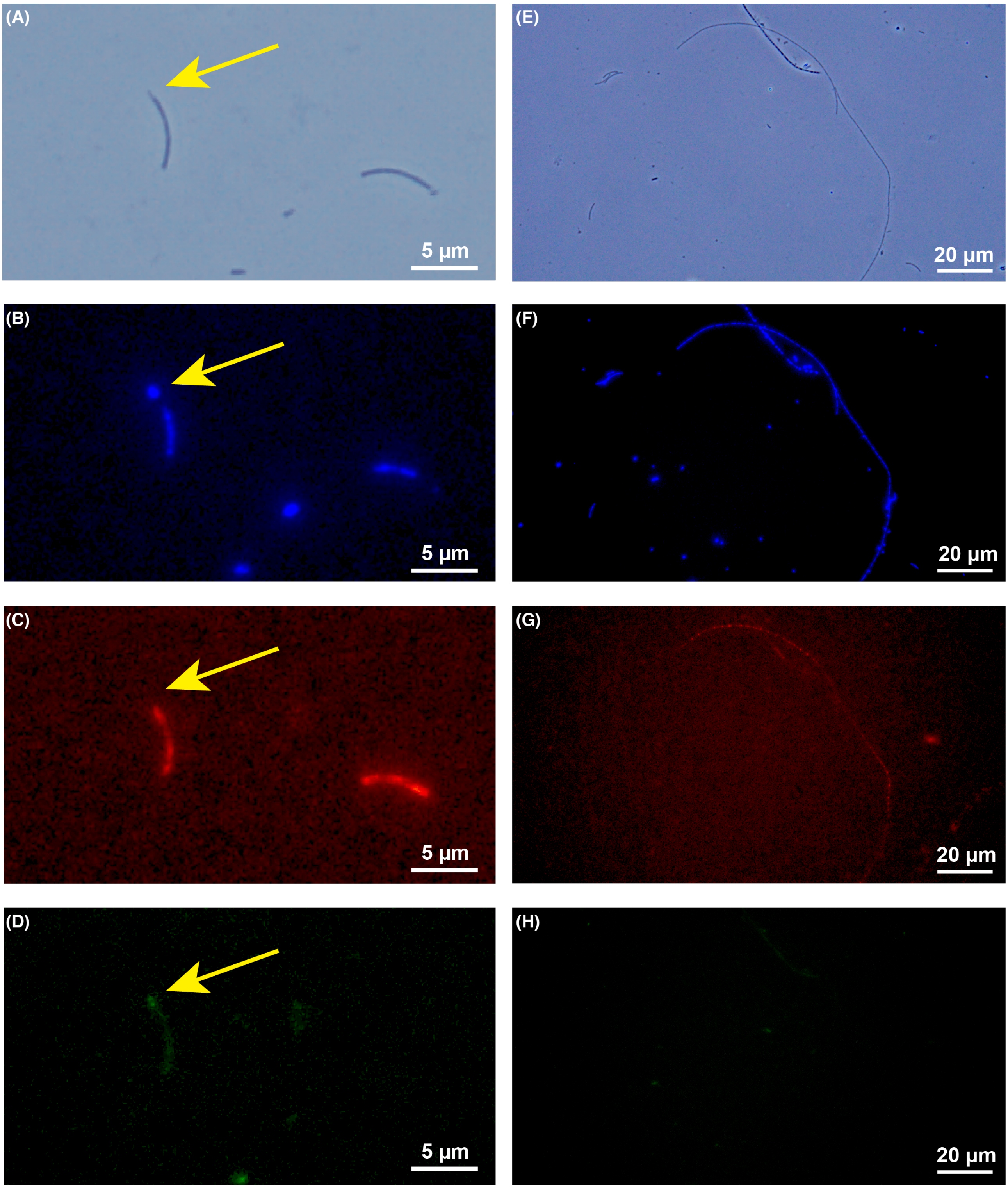

Figure S2 Micrographs of (A) and (E) Phase-contrast, (B) and (F) 4',6-diamidino-2-phenylindole dihydrochloride staining, (C), (D), (G), and (H) fluorescence *in situ* hybridization obtained from culture system A-d2-d1 (A–D) and B-d1-d1 (E–H) on cultivation days 23. (C) *Methanomicrobiales*-targeting MG1200-Cy3 probe, (D) and (H) domain Bacteria-targeting EUB338mix-FITC probe, and (G) *Methanolinea*-targeting NOBI633-Cy3 probe. Yellow arrows indicate attached small bacterial cells on the *Methanospirillum* cells.
